# How to probe the spin contribution to momentum relaxation in topological insulators

**DOI:** 10.1038/s41467-017-02420-4

**Published:** 2018-01-04

**Authors:** Moon-Sun Nam, Benjamin H. Williams, Yulin Chen, Sonia Contera, Shuhua Yao, Minghui Lu, Yan-Feng Chen, Grigore A. Timco, Christopher A. Muryn, Richard E. P. Winpenny, Arzhang Ardavan

**Affiliations:** 1 The Clarendon Laboratory, Department of Physics, University of Oxford, Oxford, OX1 3PU UK; 2 National Laboratory of Solid State Microstructures & Department of Materials Science and Engineering, Nanjing University, 210093 Nanjing, China; 3 School of Chemistry and Photon Science Institute, The University of Manchester, Manchester, M13 9PL UK

## Abstract

Topological insulators exhibit a metallic surface state in which the directions of the carriers’ momentum and spin are locked together. This characteristic property, which lies at the heart of proposed applications of topological insulators, protects carriers in the surface state from back-scattering unless the scattering centres are time-reversal symmetry breaking (i.e. magnetic). Here, we introduce a method of probing the effect of magnetic scattering by decorating the surface of topological insulators with molecules, whose magnetic degrees of freedom can be engineered independently of their electrostatic structure. We show that this approach allows us to separate the effects of magnetic and non-magnetic scattering in the perturbative limit. We thereby confirm that the low-temperature conductivity of SmB_6_ is dominated by a surface state and that the momentum of quasiparticles in this state is particularly sensitive to magnetic scatterers, as expected in a topological insulator.

## Abstract (web summary)

Magnetic scattering may profoundly modify the electronic properties of a topological insulator. Here, Nam et al. report a method enabling separation of the effects of magnetic and non-magnetic scattering by decorating the surface of topological insulators with molecules.

## Introduction

Some materials with heavy elements and strong spin–orbit coupling exhibit a bulk insulating state that is topologically distinct from traditional insulators or the vacuum; these materials are known as topological insulators (TI)^1–3^. A junction between topologically distinct insulators is always accompanied by a band crossing the Fermi energy, and a delocalised surface state at the interface whose properties reflect the nature of the insulators on each side. Spin-momentum locking, an inherent property of the dispersion of the surface state of a spin–orbit-driven TI, is a central manifestation of a time reversal (TR) symmetry protected topological surface state, and has a profound effect on the mechanisms available for quasi-particle momentum relaxation. An initial state $\left| {{\bf{k}}_{\rm {i}},{\bf{s}}_{\rm {i}}} \right\rangle$ may be perfectly back-scattered to $\left| {{\bf{k}}_{\rm {f}},{\bf{s}}_{\rm {f}}} \right\rangle = \left| { - {\bf{k}}_{\rm {i}}, - {\bf{s}}_{\rm {i}}} \right\rangle$ only by a scattering centre supplying both spin angular momentum Δ**s** = **s**_f_ − **s**_i_ and Fourier components in its spatial potential Δ**k** = **k**_f_ − **k**_i_, i.e. by a TR-symmetry-breaking (magnetic) scattering centre. In a three-dimensional TI, whose surface state is two-dimensional, $\left| {{\bf{k}}_{\rm {i}},{\bf{s}}_{\rm {i}}} \right\rangle$ may be scattered to a general final state $\left| {{\bf{k}}_{\rm {f}},{\bf{s}}_{\rm {f}}} \right\rangle$ by a non-magnetic scattering centre as long as the centre offers the appropriate spatial potential Fourier components, but this process is suppressed as the overlap between **s**_i_ and **s**_f_ decreases; this means that scattering in the small-angle limit is allowed, but large-angle scattering is restricted and back-scattering is completely forbidden. Most experimental studies of these effects have been made using spectroscopic tools^1–4^, such as angle-resolved photo-emission spectroscopy (ARPES) or scanning tunnelling microscopy (STM) in Bi-based TI. As yet, electrical transport experiments have failed to probe the phenomenon of spin-momentum locking directly.

In this article, we introduce a method of probing separately the sensitivity of a surface state to perturbative magnetic and non-magnetic scatterers, without disturbing the bulk properties. We do this by decorating the surface with scattering centres whose magnetic degrees of freedom can be engineered independently of their electrostatic structure, and observing their effect on the surface state conductivity. This provides a unique insight into spin-momentum locking in the surface quasi-particle dispersion.

## Results

### The choice of scatterers

We achieve the necessary degree of control over the magnetic scattering degrees of freedom by employing as scatterers molecular species incorporating well-defined metal clusters with controllable compositions. Study of this subject has been driven by fundamental research into nano-scale magnetism^5^, and potential applications in classical^5^ and quantum information processing^6^.

**Fig. 1:**
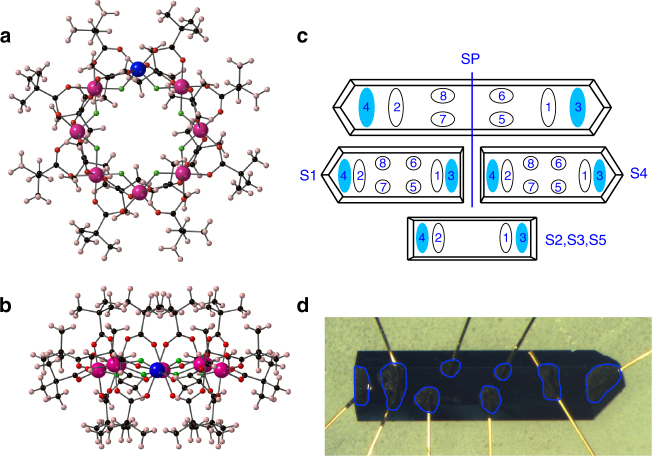
Surface modifications and sample configurations. **a**, **b** The structure of the metallic-ring molecules used to modify the TI surface state, viewed **a** perpendicular to the ring and **b** parallel to the ring. The core of eight transition metal ions (blue and magenta) in a ring geometry is stabilised by organic ligand groups to form [M_7_ZnF_8_(O_2_C^*t*^Bu)_16_]^−^(Me_2_NH_2_)^+^. The (Me_2_NH_2_)^+^ counterion is not shown. With M = Cr, the molecule is magnetic with a ground state spin of *S* = 3/2; M = Ga is perfectly diamagnetic. The non-conjugated organic ligands surround the metallic core in all directions, isolating the core from the surface onto which the molecule is sublimed. This ensures that the interaction between the metallic core and the surface is perturbative. **c** Schematic sketches of the sample and contact geometries used (filled blue ovals: current contacts; open ovals: voltage contacts). S1 and S4 were obtained by carefully breaking SP in half, and are mounted with contacts permitting measurements of both longitudinal and transverse resistance components. After characterising their pristine electrical transport characteristics, S1 was decorated with Cr_7_Zn and S4 was decorated with Ga_7_Zn. To check the reproducibility of the effects of our surface modification, we performed longitudinal resistance measurements on three further samples, S2, S3, and S5. After measurements of the pristine samples, S2 and S3 were decorated with Cr_7_Zn and S5 was decorated with Ga_7_Zn. **d** A photograph of S1 showing the hand-applied carbon-paste contacts and 25 μm gold wires. The contacts are outlined in blue for clarity

Figure 1a, b show the family of molecular magnets that we used to decorate the TI surface. Eight transition metal ions (blue and magenta) are covalently bound in a wheel-like geometry by carboxylate bridging groups, which mediate antiferromagnetic exchange interactions *J*/*k*_B_ ~ 100 K if the metal ions are magnetic. The carboxylate bridges support bulky organic groups surrounding the metallic core. A net charge of −1*e* on the metallic cluster is balanced by a singly charged counter-ion threading the ring^7,8^. We employ two members of this class, one incorporating seven Cr^3+^ ions and one Zn^2+^ ion (hereafter: Cr_7_Zn), the other incorporating seven Ga^3+^ and one Zn^2+^ (Ga_7_Zn). In these charge states, Zn and Ga are diamagnetic, and the Cr ions each carry a spin of *S* = 3/2. Thus, as a result of the antiferromagnetic interactions, Cr_7_Zn exhibits a total ground state spin of *S* = 3/2 and magnetic excitations with an energy scale of *k*_B_ × 10 K. Ga_7_Zn, composed entirely of diamagnetic constituents, is itself diamagnetic at all temperatures^7,8^.

Electron spin resonance and magnetisation experiments on bulk crystals of Cr_7_Zn (and analogue magnetic compounds Cr_7_Ni and Cr_7_Mn) reveal that neighbouring magnetic cores are electronically completely isolated and are coupled to one another only by the through-space magnetic dipole interaction^7,8^. In particular, the crystals are Van der Waals-bonded and electrically insulating; there is no electronic tunnelling between metallic cores, and therefore no exchange interaction. This implies that, when the molecules are deposited onto a conducting substrate, we expect the bulky unconjugated ligands to preclude direct electronic tunnelling between the molecular metallic core and the substrate.

Crucially for our purposes, Ga_7_Zn and Cr_7_Zn are very much alike electrostatically; they are so similar that they readily co-crystallise from solution with the two components homogeneously distributed^9^. Furthermore, they sublime into a vacuum at temperatures well below the temperature at which they decompose (in excess of ~500 K), forming an insulating layer on arrival at a room-temperature substrate^10^. There are two classes of interactions between the molecules and the substrate: electrostatic (for both Ga_7_Zn and Cr_7_Zn) and magnetic dipole (for Cr_7_Zn only). The electrostatic interaction binds the molecules to the substrate. The molecules’ dimensions are in the 1.5 nm range, so this layer introduces a potential with appreciable Fourier amplitude up to a wavevector of about 4 × 10^9^ m^−1^. The magnetic dipole interaction is present only for Cr_7_Zn because Ga_7_Zn is diamagnetic. The characteristic strength of this interaction between electronic moments on the 5 Å length scale (the separation between the magnetic core and the surface imposed by the organic ligands) is of order 5 μeV, or of order 1 GHz (in frequency units). The lack of direct tunnelling between the magnetic cores and to the substrate means that the perturbations to the substrate arise principally from the first layer of molecules deposited on the surface. The magnetic cores of the next layer of molecules are about 15 Å or further from the surface (i.e. at least three times more distant than the first layer); the inverse-cubic dependence of the dipole interaction on distance means that their coupling with the surface is weaker by more than an order of magnitude.

Since it is diamagnetic, depositing a layer of Ga_7_Zn onto a material supporting a surface state introduces a purely electrostatic scattering potential. A layer of Cr_7_Zn introduces a very similar electrostatic scattering potential, differing only in that it also supplies magnetic degrees of freedom. Thus, by comparing the change in the conducting properties of the surface state caused by the application of Cr_7_Zn with the change caused by Ga_7_Zn, we can separate the contributions of magnetic scatterers from non-magnetic (but electrostatically identical) scatterers. Owing to the spin-momentum locking exhibited by quasi-particles in the surface state of a topological insulator, magnetic scatterers, which can cause both backscattering and small-angle scattering, should be more effective in relaxing momentum than non-magnetic scatterers, which can cause only small-angle scattering.

Our approach to probing the spin-momentum locking has the advantage that it examines the response to small perturbations, in contrast with other attempts to study the effects of magnetic scattering, such as, for example, bulk doping with magnetic species which substantially modifies the material under study, or introduction of ferromagnetic materials which are themselves electrically conducting^11–17^. Our strategy of exploiting surface-deposited molecules as scatterers could be pursued using other families of isostructural but magnetically distinct molecules. For example, the family of metal phthalocyanines has both magnetic and diamagnetic members, and these compounds sublime readily. However, the highly planar structure of the phthalocyanine means that the central metal ion is in direct contact with the surface onto which it is sublimed, probably causing strong (i.e. beyond perturbative) interactions between the metal centre and the TI substrate^4^.

### The choice of TI

Since our experiment relies on the measurement of potentially small changes to the conductivity of the surface state, it is important that the TI exhibits sufficiently insulating bulk properties that the conductivity is dominated by the surface state. (This is actually rather rare in most TI that have been identified so far^3^.) This informs our choice of SmB_6_ as a TI system to study. SmB_6_ exhibits an approximately activated behaviour, with its resistivity increasing by a factor of about 10^5^ as the temperature falls from 50 K to about 4 K; below 4 K the resistivity nearly saturates^18–20^. This curious temperature dependence was not well-understood until it was recognised that the Kondo insulator bulk state has a non-trivial topology, and that the resulting surface state is responsible for the saturation of the resistivity at low temperature^21–23^. The spin–orbit-driven hybridisation between the 5*d* and 4*f* orbitals results in three spin-momentum-locked surface bands, which have been observed in angle-resolved photoemission experiments^24–26^ and confirmed as two-dimensional in magnetisation measurements^27^. While there is a lack of consensus on the exact paramters of the surface state Fermi surfaces, there is agreement that two pockets are located at the *X*-points with one smaller pocket at Γ^24–27^. Studies of non-local conductivity and sample shape and contact configuration dependences confirm the surface character of the low-temperature-conducting state^28,29^ but do not probe spin-momentum locking in the surface state.

### Experiment design

Our experiment was designed as follows. On a selection of SmB_6_ single crystals (synthesised by spontaneous nucleation from high-temperature solutions, see Methods), we made electrical contacts (using 25 μm gold wire and either graphite paste or silver-loaded conductive paste) as shown in Fig. 1c, d. We characterised the electrical transport properties of these pristine samples as a function of temperature and applied magnetic field. Onto some of the samples we sublimed in excess of a monolayer of Cr_7_Zn, and onto the remaining samples we sublimed in excess of a monolayer of Ga_7_Zn. Finally, we re-characterised the electrical transport properties of each sample. Between the characterisation of the pristine samples and the surface-modified samples, there were no changes to the samples’ geometries or electrical contact configurations. Thus any (even small) changes in the electrical transport properties can be ascribed to the effect of the introduction of the molecular scattering centres. We configured some samples for measurement of both Hall effect (*R*_*xy*_) and longitudinal resistance (*R*_*xx*_) and others for longitudinal resistance only (see Fig. 1c, d).

### Electrical transport in pristine samples

**Fig. 2:**
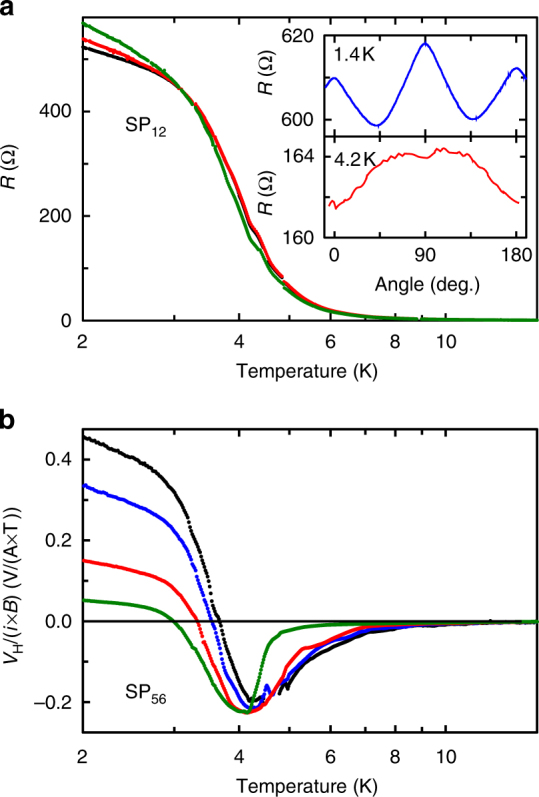
Electrical transport properties of pristine SmB_6_ samples. **a** Resistance *R*_*xx*_ as a function of temperature in magnetic fields of 0 T (black), 6 T (red), and 15 T (green). In the high-temperature regime above 5 K the bulk dominates transport; at low temperatures below about 3 K, the surface dominates. Inset: angle dependence of the 15 T magnetoresistance in the low-temperature regime (upper, blue) and at intermediate temperatures (lower, red) showing the difference in the forms of the magnetoresistance characteristic of surface-dominated and bulk-dominated transport. **b** The Hall effect *V*_H_ as a function of temperature in magnetic fields of 1 T (black), 2 T (blue), 6 T (red), and 15 T (green). The codes SP_12_ and SP_56_ indicate the particular sample and voltage contact configurations reported; see Fig. 1c for details of all samples

Figure 2 shows the electrical transport properties characteristic of pristine SmB_6_ samples. Figure 2a shows the longitudinal resistance as a function of temperature for a selection of magnetic fields (black: 0 T, red: 6 T, green: 15 T). The behaviour is consistent with previous reports: at high temperatures, electrical conduction is dominated by the bulk and the sample resistance is low; there is an intermediate temperature regime where the bulk Kondo gap is forming and the resistance increases; at temperatures below about 3 K, the resistance saturates somewhat when the sample’s conduction becomes dominated by the surface state. There is a small positive magnetoresistance in the low-temperature regime. The inset in Fig. 2a shows the dependence of the magnetoresistance on magnetic field orientation (angle-dependent magnetoresistance (ADMR) is a powerful tool for investigating the geometry of the quasi-particle dispersion, yielding, for example, detailed information about the Fermi surfaces of high *T*_c_ superconductors^30^), at 1.4 K (well into the low-temperature regime) and 4.2 K (in the intermediate temperature regime). The significant change in the form of the ADMR is characteristic of the transition from bulk-dominated conduction at high temperatures to surface-dominated conduction at 1.4 K.

Figure 2b shows the Hall effect as a function of temperature measured at a range of magnetic fields (black: 1 T, blue: 2 T, red: 6 T, green: 15 T). Again, the transition from bulk-dominated to surface-dominated transport is evident, with the Hall effect changing sign in the intermediate temperature regime.

Note that the transport coefficients in Fig. 2 are not normalised to the sample or contact geometries (i.e. we have plotted resistance not resistivity, and Hall voltage not Hall coefficient). This is because our experimental design seeks to probe changes in these quantities as we modify the surface; we are less interested in the absolute values of the transport coefficients, which are, as we shall see below, difficult to evaluate accurately for our as-grown and somewhat irregular crystal geometries.

### The effect of surface modification on electrical transport

**Fig. 3:**
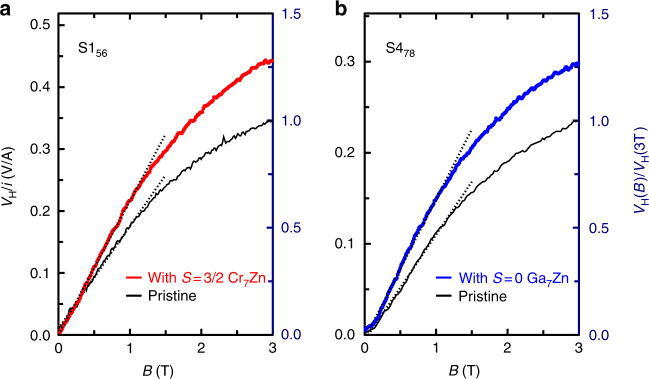
The change in the low-temperature Hall effect under the influence of surface modification. **a**, **b** Black curves: the Hall voltage on pristine samples; **a** red curve: the Hall voltage after application of magnetic Cr_7_Zn; **b** blue curve: Hall voltage after the application of non-magnetic Ga_7_Zn. The temperature is 1.4 K. Dashed lines are linear fits to the low-field behaviour, from which the carrier densities are derived. The left-hand vertical axes indicate the absolute value of the Hall resistance measured; the right-hand axes indicate the Hall voltage normalised to its pristine value at 3 T, demonstrating that the relative change in Hall effect is the same for both surface modifications. Thus the change in carrier density is the same for both surface modifications, indicating that Cr_7_Zn and Ga_7_Zn offer equivalent electrostatic perturbations

Figure 3a, b show the low-temperature Hall resistances as a function of magnetic field for two samples (S1_56_ and S4_78_) before any surface modification (pristine, black) and after surface modification with (a) Cr_7_Zn (red) and (b) Ga_7_Zn (blue). In the pristine state, the Hall effects are characteristic of a single carrier, indicating that one of the surface bands (probably the one centred on the X-point) dominates by virtue of its high mobility. In this limit, for a 2D conductor, the Hall voltage *V*_H_ in a small magnetic field *B* is related to the carrier density *n* as *V*_H_ = *iB*/*ne*, where *i* is the current flowing between the Hall contacts and *e* is the charge on the carriers. Naively, this expression yields surface state carrier densities of *n*_pristine_ = (3.78 ± 0.03) × 10^15^ cm^−2^ and *n*_pristine_ = (5.38 ± 0.03) × 10^15^ cm^−2^ for samples S1_56_ and S4_78_, respectively. Given that these two measurements were made on a common face of what was originally a single crystal, we expect the same carrier density for each. The difference reflects the imperfect geometry of the measurement; the current actually includes components flowing beneath the contacts and elsewhere in the surface state rather than only between the Hall contacts (leading to an overestimate of *n*), and the positioning of the hand-applied contacts is not identical for the two crystals.

Attempting to correct these imperfections inevitably introduces large errors associated with uncertainties in the geometry. As long as the geometries of the samples remain fixed, however, small relative changes in transport coefficients (for example, caused by our surface modification) can be measured very precisely. This highlights the motivation for our experimental strategy of characterising each of our samples in the pristine condition before applying the surface modification; it constitutes a calibration of each sample geometry, before introducing the perturbative change that we seek to measure.

The Hall resistances that we measure after surface modification yield nominal carrier densities of *n*_Cr7Zn_ = (2.89 ± 0.01) × 10^15^ cm^−2^ and *n*_Ga7Zn_ = (4.07 ± 0.02) × 10^15^ cm^−2^ for the Cr_7_Zn- and Ga_7_Zn-decorated samples, respectively. These nominal values are subject to exactly the same geometrical errors as the pristine samples, so the significant quantities that we can extract are ratios of the carrier densities before and after surface modification, *n*_Cr7Zn_/*n*_pristine_ = 0.766 ± 0.007 and *n*_Ga7Zn_/*n*_pristine_ = 0.757 ± 0.006. We may draw two important conclusions from the observation of the sensitivity of the Hall effect to surface modification.

First, the fact that the deposition of a very thin layer of an insulating dielectric material has a significant effect on the electrical transport properties offers further confirmation that, in this temperature regime, the electrical transport is dominated by a surface state. (This supports earlier conclusions drawn from completely independent experimental probes, such as, for example, non-local electrical transport experiments^28,29^.) We note here that, while the existence of the surface state is topologically protected, its occupation is not. The effect of the surface modification is to gate electrostatically the surface state. (This may help to explain the discrepancies between the sizes of the surface-state Fermi surfaces reported in the literature; it is not surprising that experiments conducted in different environments^24–27^ will yield different Fermi surface sizes.)

Second, there is no significant difference in the change in carrier density induced by each of our surface modifications (both change the carrier density by a factor of 0.76). This is a reflection of the similarity between the two molecules, and confirms that they each introduce the same electrostatic perturbation.

We are now in a position to investigate, via the changes in the longitudinal resistivity, how our surface modifications affect the surface state mobility and hence the momentum scattering rate. The resistivity, *ρ*, is given by *ρ* = (*neμ*)^−1^ where the mobility *μ* is proportional to the scattering time *τ*.

**Fig. 4:**
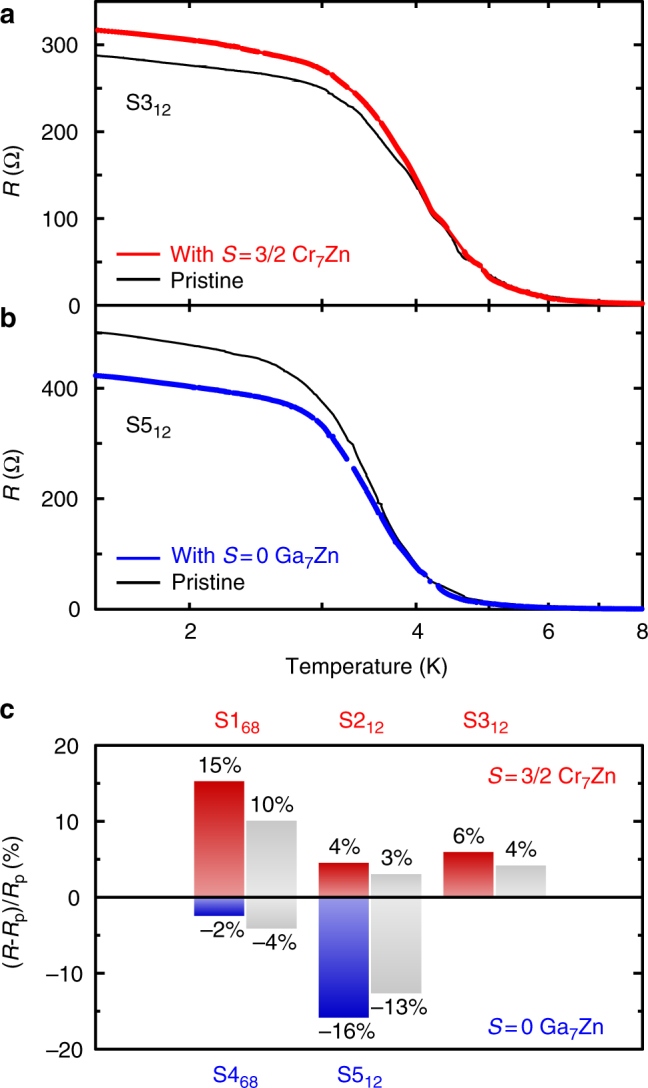
The change in resistance as a function of temperature under the influence of surface modification. The effect of the magnetic Cr_7_Zn (**a**, red) is to perturbatively increase the resistance at low temperature and Ga_7_Zn (**b**, blue) causes a perturbative decrease in resistance at low temperatures. At high temperatures where the electrical transport is dominated by the bulk, surface modification has no effect. **c** Graphical representation of the deviation of zero-magnetic-field resistance caused by surface decoration for all samples. The coloured (grey) bars represent the resistance changes at 1.4 K (4.2 K). The measured values of resistance are reproduced in Table 1

Figure 4 shows the changes in sample resistance as a function of temperature (with no applied magnetic field) when (a) Cr_7_Zn and (b) Ga_7_Zn are introduced. At high temperatures, where the conductivity is dominated by the bulk, there is no sensitivity to the surface modification; the resistances of the unmodified and modified samples overlay. In the low-temperature regime, where the conduction is through the surface state, there are significant changes, and, unlike the Hall effect, the changes in resistance do depend on whether the scattering centres are magnetic (Cr_7_Zn) or non-magnetic (Ga_7_Zn).

Across all samples studied, we find that the effect of the non-magnetic Ga_7_Zn surface modification is to perturbatively decrease the surface state resistance, whereas the effect of the magnetic Cr_7_Zn is to perturbatively increase the surface state resistance (see Fig. 4c and Table 1). Given that both modify the carrier density in the same way, we conclude that mobilities are systematically lower in the presence of Cr_7_Zn than in the presence of Ga_7_Zn.

**Table 1:** Absolute changes of zero-magnetic-field resistances caused by surface decoration

**a**. The effect of decoration with Cr_7_Zn, *B* = 0 T						
	*T* = 1.4 K			*T* = 4.2 K		
	S1_68_	S2_12_	S3_12_	S1_68_	S2_12_	S3_12_
Without	143.62 Ω	598.74 Ω	288.42 Ω	48.64 Ω	141.85 Ω	111.80 Ω
With	165.40 Ω	625.33 Ω	305.30 Ω	53.11 Ω	146.07 Ω	116.37 Ω
Δ*R*	+21.78 Ω	+26.59 Ω	+16.88 Ω	+4.47 Ω	+4.22 Ω	+4.57 Ω

**b**. The effect of decoration with Ga_7_Zn, *B* = 0 T				
	*T* = 1.4 K		*T* = 4.2 K	
	S4_68_	S5_12_	S4_68_	S5_12_
Without	110.22 Ω	502.4 Ω	33.32 Ω	50.28 Ω
With	107.61 Ω	423.33 Ω	31.98 Ω	44.00 Ω
Δ*R*	−2.61 Ω	−79.07 Ω	−1.34 Ω	−6.28 Ω

**Fig. 5:**
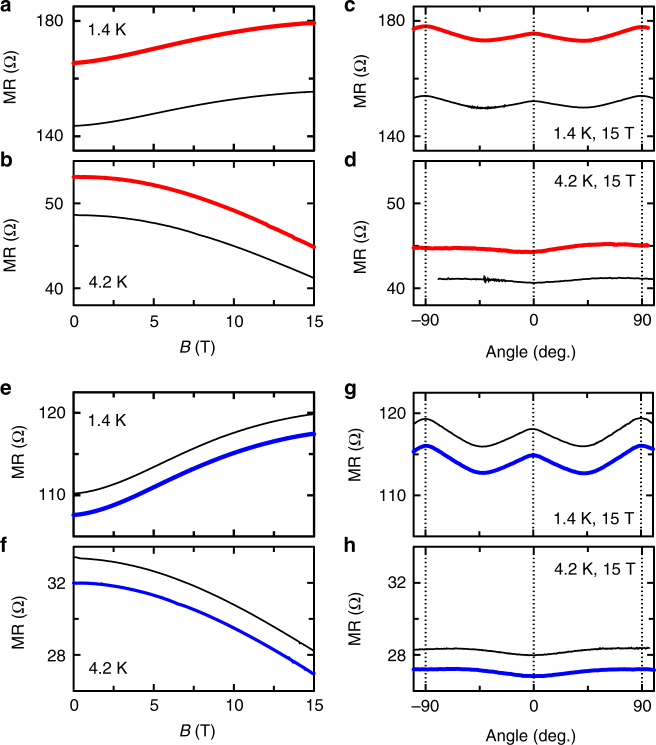
The change in magnetoresistance under the influence of surface modification. **a**–**d** The change in magnetoresistance induced in sample S1_68_ on application of Cr_7_Zn as a function of magnetic field at **a** 1.4 K and **b** 4.2 K, and as a function of orientation in high magnetic field at **c** 1.4 K and **d** 4.2 K. Black traces represent pristine behaviour; red traces follow the surface modification. **e**–**h** The change in magnetoresistance induced in sample S4_68_ on application of Ga_7_Zn as a function of magnetic field at **e** 1.4 K and **f** 4.2 K, and as a function of orientation in high magnetic field at **g** 1.4 K and **h** 4.2 K. Black traces represent pristine behaviour; blue traces follow the surface modification. For both surface modifications, the changes in magnetoresistance are small and the form of the magnetoresistance as a function of magnetic field and orientation is preserved, confirming that the changes introduced by our surface modifications are perturbative and do not modify the Fermi surface geometry

Figure 5 shows how the magnetoresistance and the ADMR change on application of (a–d) magnetic Cr_7_Zn and (e–h) non-magnetic Ga_7_Zn. The magnetic scatterers cause a small increase in the resistance of the surface state. The increase is multiplicative with a factor that is independent of both magnetic field strength and orientation; the magnetic scattering is effective over the whole parameter space that we studied, owing to the random orientations of the molecular scatterers (and their anisotropy axes) and the existence of intramolecular magnetic excited states. The non-magnetic scatterers cause a small multiplicative decrease in resistance. The fact that the form of the magnetoresistance and the ADMR is unchanged in both cases confirms that the effect of both types of scatterers is indeed perturbative. Thus, although the surface modifications perturb the filling of the surface state and the quasi-particle scattering, they do not substantially alter the geometry of the Fermi surface.

## Discussion

In samples S1 and S4, for which we have both Hall effect and longitudinal resistance data, we can extract quantitative estimates of the changes in electrical transport properties for each surface modification. Since the contact and sample geometries are fixed throughout the experiment, the change in resistance on surface modification is directly related to the change in mobility. Thus, for sample S4 at 1.4 K, *R*_Ga7Zn_/*R*_pristine_ = *ρ*_Ga7Zn_/*ρ*_pristine_ = *n*_pristine_*μ*_pristine_/*n*_Ga7Zn_*μ*_Ga7Zn_ = 0.98. Using the ratio of carrier densities obtained from the Hall effect, we can derive the ratio of mobilities: *μ*_Ga7Zn_/*μ*_pristine_ = 1.35.

Thus, we find that the mobility increases on surface modification with non-magnetic Ga_7_Zn. In isolation, we would expect the introduction of an electrostatic scattering potential to decrease the mobility (though in the presence of spin-momentum locking, back-scattering is suppressed and only small-angle scattering is enhanced). However, the mobility is expected to depend also on the filling of the surface state. For example, in graphene the mobility increases dramatically as band filling decreases^31^, and significant dependences of mobility on band filling are observed in a range of other two-dimensional systems^32^.

For sample S1 at 1.4 K, we measure *R*_Cr7Zn_/*R*_pristine_ = *n*_pristine_*μ*_pristine_/*n*_Cr7Zn_*μ*_Cr7Zn_ = 1.15. Again accounting for the ratio of the carrier densities, we find that the magnetic Cr_7_Zn scatterers change the mobility by a factor *μ*_Cr7Zn_/*μ*_pristine_ = 1.14. Since the samples S1 and S4 were two pieces of a single crystal, we expect the mobilities in the pristine state to be equal, so we can compare the mobilities in the presence and absence of magnetic degrees of freedom: *μ*_Cr7Zn_/*μ*_Ga7Zn_ = 0.84. We reiterate here that the enhancement of the scattering caused by Cr_7_Zn over Ga_7_Zn can arise only from the presence of the magnetic degree of freedom; all other factors are normalised away by design in our experiment.

Our observations lead to three robust conclusions. First, we can control electrostatic and magnetic perturbations to a surface state independently by decorating the surface with appropriately chosen molecular scatterers: Ga_7_Zn introduces a random electrostatic potential only, while Cr_7_Zn introduces an identical electrostatic potential and also coupled magnetic degrees of freedom. Second, the fact that these delicate surface modifications substantially affect the low-temperature electrical conduction mechanisms in SmB_6_ offers independent experimental confirmation that the conductivity in the low-temperature regime is indeed dominated by a surface state in this material. Third, and most significant, the relative changes in zero-field resistance on surface decoration confirm that the magnetic scattering centres are significantly more effective at relaxing quasi-particle momentum, consistent with the predictions for a TR-protected TI surface state. Our results demonstrate the effectiveness of an experimental method for modifying a surface state by perturbing it in a controlled way (both magnetically and electrostatically). We believe that this offers a generally applicable experimental approach to separating the roles of magnetic and electrostatic scatterers in surface states.

## Methods

### SmB_6_ sample preparation

High-quality single crystals of SmB_6_ were grown by spontaneous nucleation from high-temperature solutions, using Al as solvent. The starting materials were Sm, B, and Al with a purity of 99.99%. The molar ratio of solute to solvent was 1:10. The mixture was heated to 1500 °C and held at this temperature for 24 h to homogenise the solution in an argon atmosphere. After superheating, the melt was cooled to 1450 °C quickly and then slowly cooled to 700 °C. After this, the melt was cooled to room temperature naturally. Single crystals with dimensions of up to 4 mm × 0.8 mm × 0.4 mm were obtained by dissolving the Al flux using hydrochloric acid.

### Cr_7_Zn and Ga_7_Zn preparation

[M_7_ZnF_8_(O_2_C^*t*^Bu)_16_]^−^(Me_2_NH_2_)^+^ with M = Cr (Cr_7_Zn) and M = Ga (Ga_7_Zn) were synthesised using the literature methods^7,9^. Cr_7_Zn and Ga_7_Zn were deposited in a manner similar to that previously reported^10^. The SmB_6_ crystals were held at pressures of about 2 × 10^−8^ mbar, at a distance of 10 cm from an ohmically heated crucible containing a crystalline powder of either Ga_7_Zn or Cr_7_Zn. The molecular sublimation occurs in the temperature range of 180–230 °C. The deposition rate was monitored using a quartz crystal microbalance, and we deposited in excess of a single monolayer on each sample. We confirmed the presence and morphology of the molecular layer using atomic force microscopy (see Supplementary Figs. 1 and 2).

### Electrical transport measurements

We used the low-frequency lock-in technique to measure the transverse and longitudinal voltages with currents of 10 μA at 13.7 Hz. In this range, all voltages were proportional to the applied current and independent of frequency. All measurements in magnetic fields were performed both in negative and positive fields. Magnetoresistance data were obtained by averaging positive and negative field traces; Hall effect data were obtained by halving the difference between positive and negative field traces. The ADMR measurements were performed by rotating the samples about their longest axis, such that the magnetic field was rotated in the *b*–*c* plane. The current direction was at all times perpendicular to the magnetic field.

To maintain the temperature stability, samples and the rotational mechanism were fully immersed either in liquid helium at 4.2 K or in superfluid helium at 1.4 K for the duration of magnetic fields or angle sweeps. Temperature stability is critical in this material because in some temperature ranges, the resistance changes rapidly with temperature.

### Data availability

The data that support the findings of this study are available from the authors on reasonable request.

## Electronic supplementary material

Supplementary Information
