# Author Correction: How to probe the spin contribution to momentum relaxation in topological insulators

**DOI:** 10.1038/s41467-018-03331-8

**Published:** 2018-02-15

**Authors:** Moon-Sun Nam, Benjamin H. Williams, Yulin Chen, Sonia Contera, Shuhua Yao, Minghui Lu, Yan-Feng Chen, Grigore A. Timco, Christopher A. Muryn, Richard E. P. Winpenny, Arzhang Ardavan

**Affiliations:** 1 The Clarendon Laboratory, Department of Physics, University of Oxford, OX1 3PU Oxford, UK; 2 National Laboratory of Solid State Microstructures & Department of Materials Science and Engineering, Nanjing University, 210093 Nanjing, China; 3 School of Chemistry and Photon Science Institute, The University of Manchester, M13 9PL Manchester, UK

Correction to: *Nature Communications*(2017) 10.1038/s41467-017-02420-4, published online 04 January 2018

The original version of this Article contained an error in the spelling of the author Benjamin H. Williams, which was incorrectly given as Benjamin H. Willams. This has now been corrected in both the PDF and HTML versions of the Article.

