# Supplementary material for: How to probe the spin contribution to momentum relaxation in topological insulators
Source: Nat Commun. 2018 Jan 4;9:56. doi: 10.1038/s41467-017-02420-4 (PMC5754345; doi:10.1038/s41467-017-02420-4)
Supplement: Supplementary file 1 — Supplementary Information [file 41467_2017_2420_MOESM1_ESM.pdf]

Supplementary Note

Atomic force microscopy characterisation of pristine and modified SmB_6 surfaces

In order to confirm the presence of the molecular layer after deposition, we characterised both pristine and modified SmB_6 surfaces using atomic force microscopy (AFM).

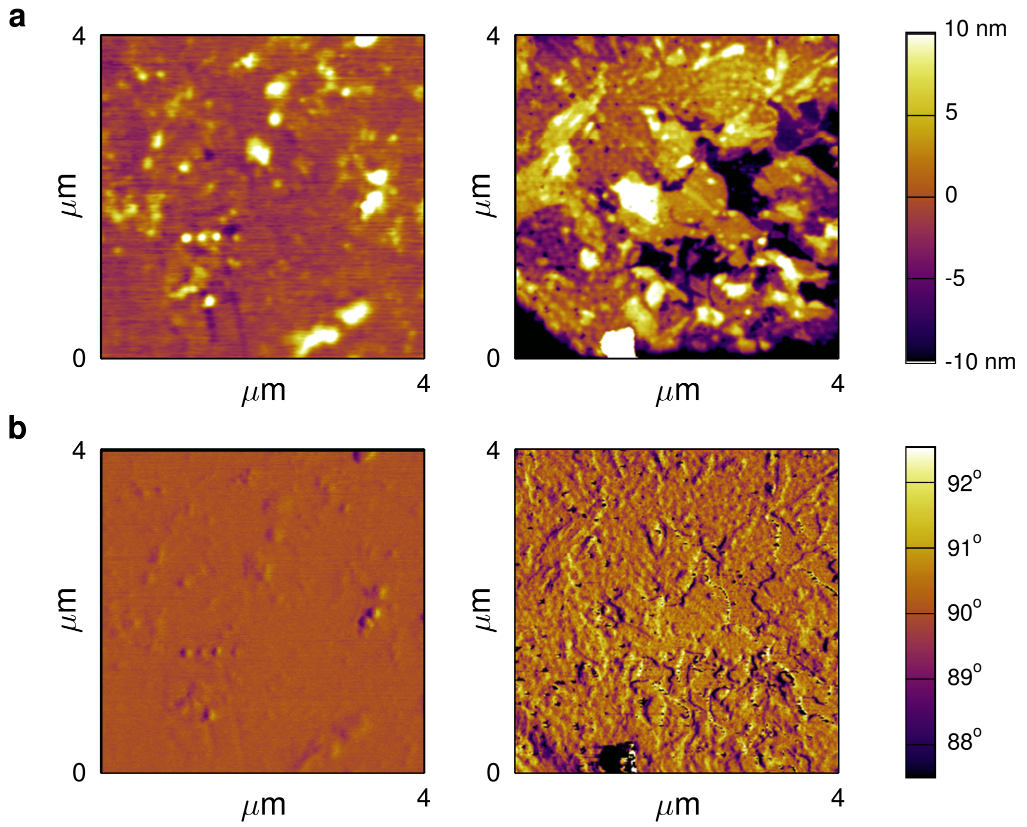

Supplementary Figure 1| AFM characterisation of pristine and modified SmB_6 surfaces. **a,** Topography images and **b,** phase images of pristine (left) and modified (right) SmB_6 surfaces.

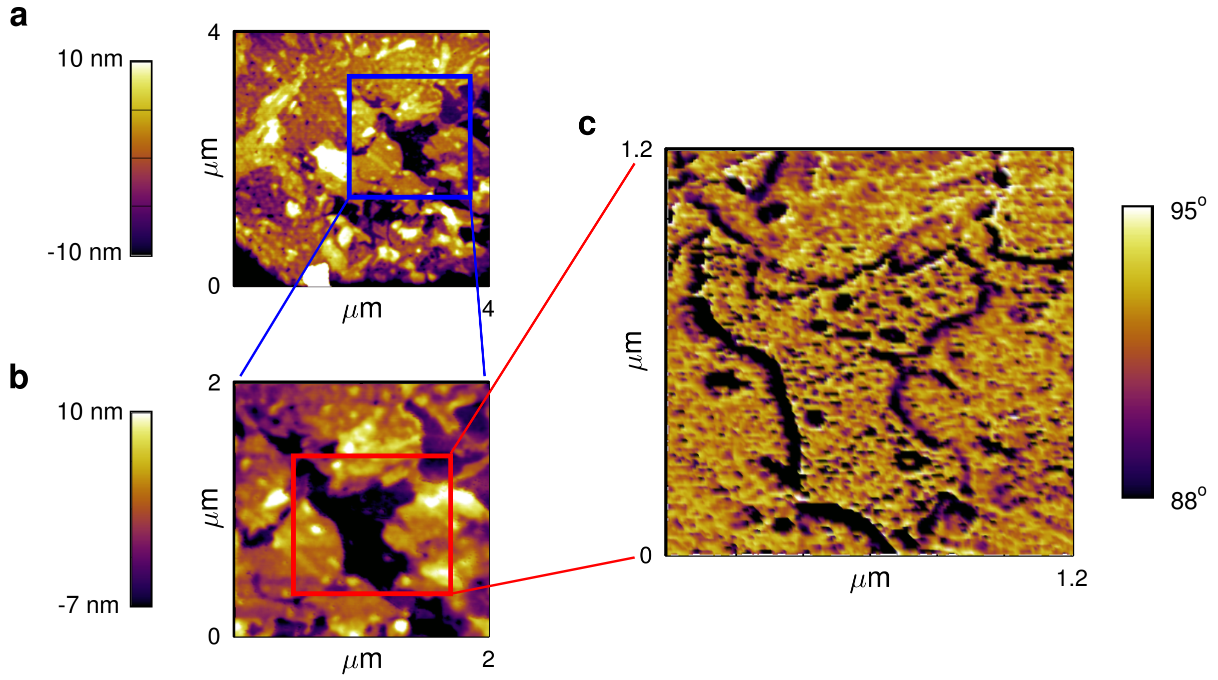

Supplementary Figure 2| AFM characterisation of modified SmB_6 surfaces. a, b, AFM topography images of a particular area of modified SmB6 surface on several scales. **c**, Phase image of the same region as the lower left panel.

Supplementary Figure 1a shows topographic images of pristine (left) and modified (right) surfaces. The pristine surface exhibits some roughness, but the morphology of the surface changes significantly following surface modification, consistent with the presence of a molecular layer. Supplementary Figure 1b shows phase images of the same regions. The pristine surface is uniformly stiff, as expected for a SmB_6 surface. The modified surface shows the much greater variation of phase characteristic of a soft and amorphous surface, as expected for a Van der Waals-bonded molecular layer.

Supplementary Figures 2a and 2b show topographic images of a modified surface at successively smaller length scales. There is structure at all length scales visible. Supplementary Figure 2c shows a phase image of the smallest area (phase of the second eigenmode).
